# Baseline cognitive insight moderates the efficacy of metacognitive training for psychosis: an individual participant data analysis

**DOI:** 10.3389/fpsyg.2026.1934181

**Published:** 2026-10-27

**Authors:** Adrien Goncalves, Susana Ochoa, Steffen Moritz, Łukasz Gawęda, Vanessa Acuña, Caroline König, Maria Lamarca, Fabrice Berna

**Affiliations:** 1 INSERM, UMR_S 1329: Strasbourg Translational Neuroscience & Psychiatry, Strasbourg, France; 2 École doctorale des sciences de la vie et de la santé: ED 414, Université de Strasbourg, Strasbourg, France; 3 Parc Sanitari Sant Joan de Déu, Sant Boi de Llobregat, Barcelona, Spain; 4 Grup MERITT, Institut de Recerca Sant Joan de Déu, Esplugues de Llobregat, Barcelona, Spain; 5 Consorcio de Investigación Biomédica en Red de Salud Mental (CIBERSAM), Instituto de Salud Carlos III, Madrid, Spain; 6 Klinik und Poliklinik für Psychiatrie und Psychotherapie, Universitätsklinikum Hamburg-Eppendorf, Hamburg, Germany; 7 Experimental Psychopathology Lab, Institute of Psychology, Polish Academy of Sciences, Warsaw, Poland; 8 Departamento de Psiquiatría, Escuela de Medicina, Facultad de Medicina, Universidad de Valparaíso, Valparaíso, Chile; 9 Soft Computing Research Group at Intelligent Data Science and Artificial Intelligence Research Center, Universitat Politècnica de Catalunya, Barcelona, Spain; 10 Departament de Psicologia Clínica i de la Salut, Facultat de Psicologia, Universitat Autònoma de Barcelona, Bellaterra, Cerdanyola del Vallès, Barcelona, Spain; 11 Faculté de Médecine, Université de Strasbourg, Strasbourg, France; 12 Hôpitaux universitaires de Strasbourg, Strasbourg, France

**Keywords:** Beck cognitive insight scale, cognitive insight, individual participant data, metacognitive training, psychosis

## Abstract

**Background:**

Metacognitive Training (MCT) for psychosis is designed to target metacognitive processes, yet evidence for its effects on cognitive insight remains inconclusive, owing primarily to the limited methodological quality of available trials. A potential explanation lies in baseline heterogeneity, with a substantial proportion of participants presenting high baseline cognitive insight, thereby limiting observable change.

**Methods:**

This study used harmonized individual participant data from the PERMEPSY database, including 614 participants with schizophrenia spectrum disorders from 13 international studies. Cognitive insight was assessed using the Beck Cognitive Insight Scale (BCIS), including Self-Reflectiveness (SR), Self-Certainty (SC), and a composite index (CI). Baseline moderation effects were examined using ANCOVA models with a continuous Group × Baseline interaction term, complemented by stratified ANOVAs to visualize the interaction pattern and estimate within-stratum effect sizes.

**Results:**

ANCOVA models revealed significant Group × Baseline interactions across all BCIS dimensions, indicating that baseline cognitive insight moderated treatment response. Stratified ANOVAs yielded convergent results for SR and SC. Effect sizes followed a consistent gradient, with larger improvements observed among participants with lower baseline insight. Notably, 26.5% of participants already scored in the high-insight category at baseline, suggesting that ceiling effects may have partially obscured treatment effects at the group level in previous trials.

**Conclusion:**

MCT appears to improve cognitive insight, particularly among participants with greater baseline impairment. These findings suggest that baseline heterogeneity is a key determinant of MCT efficacy and may account for previously inconsistent results.

## 1 Introduction

Metacognitive Training (MCT) for psychosis is a low-intensity psychoeducational program derived from cognitive behavioral therapy. It specifically targets metacognitive processes through raising participants’ awareness of cognitive biases implicated in the formation and maintenance of positive psychotic symptoms. A central objective of the program is to reduce overconfidence in participants’ judgments and to plant the seeds of doubt regarding their cognitive distortions (Moritz et al., 2025). MCT is available in both group and individualized formats and has been translated into 39 languages. It is recommended by several major health organizations, including the Royal Australian and New Zealand College of Psychiatrists (Galletly et al., 2016), the German Association for Psychiatry, Psychotherapy and Psychosomatics (DGPPN, 2019; Hasan et al., 2026), and the World Health Organization (WHO, 2023). These recommendations are grounded in a substantial body of evidence. A recent systematic meta-review pooling eight meta-analyses reported significant effects of MCT on delusions, positive symptoms, total psychotic symptoms, hallucinations, and negative symptoms (Meinhart et al., 2025). A systematic review applying the World Federation of Societies of Biological Psychiatry grading framework further confirmed strong recommendations (grade 1) for positive symptoms, delusions, and total psychotic symptoms, as well as limited recommendations (grade 2) for several secondary outcomes at follow-up (Goncalves et al., 2025). However, the same review was only able to attribute a grade 4 recommendation for metacognition as a post-treatment outcome, reflecting an evidence base that remains methodologically limited, owing primarily to high risk of bias and inconsistent findings across the most rigorous randomized controlled trials available in this domain. This gap is particularly noteworthy given that metacognitive functioning constitutes one of the primary theoretical targets of the MCT program.

Metacognition, broadly defined as the capacity to monitor and regulate one’s own cognitive processes (Flavell, 1976), is a multifaceted construct that plays a central role in the pathogenesis and maintenance of psychotic symptoms (Seow et al., 2021; Abramovitch et al., 2021; Gawęda et al., 2024). A key distinction in the literature separates on-line metacognition, which involves real-time monitoring during task execution, from off-line metacognition, which refers to declarative knowledge about one’s own cognitive functioning assessed independently of performance (Dörrenbächer-Ulrich et al., 2021; Lehmann et al., 2022; Fleming, 2024). Within this framework, cognitive insight represents a core off-line metacognitive dimension, operationalized as the ability to reflect upon and critically evaluate one’s own beliefs and interpretations, and to remain open to corrective feedback (Riggs et al., 2012; Díaz-Cutraro et al., 2026). The Beck Cognitive Insight Scale (BCIS; Beck, 2004) is the most widely used instrument to assess cognitive insight in participants with schizophrenia spectrum disorders, comprising two subscales: Self-Reflectiveness (SR), which captures the tendency to acknowledge the fallibility of one’s beliefs and consider alternative interpretations, and Self-Certainty (SC), which reflects the degree of certainty in one’s judgments and resistance to correction. A composite index (CI) provides an overall measure of cognitive insight, with higher values indicating better insight (Penney et al., 2019). Beyond its conceptual relevance, cognitive insight also carries direct clinical significance since lower SR predicts greater symptom severity 4 years later (O’Connor et al., 2017), and metacognitive functioning more broadly, including cognitive insight, has been associated with poorer functional outcome in schizophrenia spectrum disorders (Davies and Greenwood, 2020). Moreover, across MCT efficacy studies, the BCIS has been the most systematically used measure of off-line metacognitive functioning, rendering it the most appropriate instrument for synthesizing evidence on this outcome (Goncalves et al., 2025). Taken together, cognitive insight represents a clinically meaningful outcome, and not merely a theoretical target of MCT.

Despite this theoretical alignment, the existing literature on MCT’s effects on cognitive insight remains highly heterogeneous and difficult to interpret. A broad systematic review and meta-analysis of psychological interventions targeting cognitive biases reported significant effects on a composite measure of insight with modest effect sizes (*Hedges’ g* = 0.35; Sauvé et al., 2020); however, this estimate should be interpreted cautiously, as it pooled both cognitive and clinical insight measures across studies of varying designs, including non-randomized trials. A more specific meta-analysis restricted to six RCTs examining MCT’s effects on cognitive insight reported a small-to-medium effect on SR (*d* = 0.46), a small effect on SC (*d* = −0.23), and a large effect on the CI (*d* = 1.11), alongside evidence of substantial heterogeneity (Lopez-Morinigo et al., 2020). These effect sizes appear to contrast with the grade 3 post-treatment recommendation assigned to a composite insight outcome combining clinical and cognitive insight, but predominantly based on cognitive insight measures (Goncalves et al., 2025). This low grade primarily reflected the poor methodological quality of the available randomized controlled trials and the inconsistency of their findings across studies. Another systematic review focusing on early schizophrenia further underscored this complexity, reporting mixed results for both CBT and MCT on cognitive insight dimensions and concluding that the current evidence base remains too limited to draw firm conclusions (Houdebine et al., 2025). This pattern echoes findings from a recent meta-analysis showing that even cognitive behavioral interventions explicitly designed to target cognitive biases in schizophrenia spectrum disorder produce inconsistent and limited effects on these very mechanisms (Massicotte et al., 2026), suggesting that the difficulty of demonstrating reliable changes in metacognitive outcomes may reflect a broader methodological and measurement challenge in the field.

A critical but underexplored explanation for this variability may lie in the characteristics of the samples enrolled in previous MCT trials. Indeed, cognitive insight has consistently been examined as a secondary outcome in MCT efficacy studies, with no minimum baseline thresholds imposed at inclusion. As a result, a non-negligible proportion of enrolled participants may already present with high levels of cognitive insight prior to treatment, mathematically constraining the potential for improvement. Even an effective intervention would be expected to produce little observable change at the group level under such conditions, a phenomenon known as a floor or ceiling effect, depending on the direction of the scale (Hegedus and Moody, 2010; Andrade, 2021). Although this mechanism has, to our knowledge, never been tested for the BCIS in the psychosis literature, it echoes findings from a large meta-analysis of psychotherapy trials for depression (387 RCTs, *n* = 47,315), where response rates increased with baseline severity in treatment arms while remaining stable in control arms (Cuijpers et al., 2026). More directly, this mechanism has a direct parallel in the positive symptom literature: when participants with subclinical baseline symptom levels are enrolled in MCT trials without minimum severity thresholds, the resulting floor effect attenuates observed treatment effects at the group level, while individual participant data reveal progressively stronger effects as baseline severity increases (Goncalves et al., under review). Whether a comparable moderation pattern exists for cognitive insight as measured by the BCIS has not yet been examined.

The present study addressed this question using harmonized individual participant data from the PERMEPSY database, an international dataset integrating retrospective data from multiple studies of MCT for schizophrenia spectrum disorders (König et al., 2025a). This approach was selected because it allows for continuous moderation analyses that preserve the full distribution of baseline scores, thereby overcoming the limitations of aggregate-level meta-analytic methods in detecting interaction effects (Riley et al., 2023). The primary aim was to examine whether baseline cognitive insight, as measured by the BCIS, moderates the efficacy of MCT on post-treatment changes in SR, SC, and CI. We hypothesized that baseline cognitive insight would moderate treatment response, such that participants with the most impaired cognitive insight at baseline would derive greater benefit from MCT. We further predicted that a non-negligible proportion of participants would already score in the high cognitive insight category at baseline (CI ≥ 10; Penney et al., 2019), and that this distributional characteristic would constitute a partial explanation for the inconsistency of effects reported in the existing literature.

## 2 Methods

### 2.1 Database and participants

Data were derived from the PERMEPSY database (König et al., 2025a), collected as part of the European ERAPERMED project “Towards a Personalized Medicine Approach to Psychological Treatment of Psychosis.” This database integrates retrospective individual participant data from individuals diagnosed with a schizophrenia spectrum disorder who underwent MCT across 22 international studies. However, the original harmonization pipeline retained only MCT participants, excluding control conditions; as the present analysis also required control participants where available, raw datasets from contributing studies were retrieved and independently re-standardized, regardless of whether the original study had included its own control condition. In addition, the BCIS had not been administered as an outcome measure in all 22 contributing studies. Fourteen of the 22 studies had administered the BCIS at baseline (see Supplementary Table 1), but one of these was excluded following data verification, owing to inconsistencies in BCIS subscale scores that could not be reliably corrected. The remaining 13 studies were retained for the present analysis and only participants with pre-treatment scores on at least one subscale of the BCIS were retained, yielding a sample of 614 individuals. Participants were allocated either to the MCT condition (comprising the original group-based MCT, the individualized MCT+ format, and the MCT Young Version), or to control conditions (comprising treatment as usual, psychoeducation, cognitive remediation, and occupational treatment). Standardization involved recoding response formats into uniform numerical scores, verifying score ranges against original scale guidelines, and computing the CI score as the arithmetic difference between SR and SC (CI = SR − SC). For one contributing study that had administered an extended 30-item version of the BCIS (Moritz et al., 2011), the research team was contacted to identify the items corresponding to the standard 15-item scale, which were subsequently extracted and scored accordingly.

### 2.2 Inclusion criteria

Participants were included if they: (1) were at least 16 years old; (2) had a diagnosis of a schizophrenia spectrum disorder; and (3) had at least one BCIS subscale score available at baseline. Exclusion criteria included severe organic brain dysfunction or a learning disability.

### 2.3 Intervention

MCT for psychosis is a structured, psychoeducational intervention targeting cognitive biases associated with psychotic symptoms, including jumping-to-conclusions bias and overconfidence in memory errors (Moritz et al., 2025). Across the 13 studies contributing to the present analysis, participants received MCT under heterogeneous delivery formats: group-based MCT was delivered over 8, 10, or 16 sessions depending on the study; individual MCT+ was delivered over 4, 8, or 10 sessions; and one further study used the MCT Young Version, delivered over 16 sessions (Ussorio et al., 2016; Supplementary Table 1). Because MCT format was determined by study protocol and did not vary within any contributing study, its effect could not be meaningfully examined as a moderator independently of study-level differences. Despite these variations in delivery, all versions share a common therapeutic framework aimed at promoting metacognitive awareness of cognitive biases, and were therefore grouped together in all analyses. All MCT versions are available at www.uke.de/mct.

### 2.4 Outcome measures

The primary outcomes were pre-to-post-treatment changes in the two subscales of the BCIS (Beck, 2004), a 15-item self-report questionnaire assessing cognitive insight. The SR subscale captures the tendency to acknowledge fallibility in one’s own beliefs and to consider alternative interpretations (range 0–36), with higher scores reflecting greater self-reflectiveness. The SC subscale reflects the degree of certainty in one’s own judgments and resistance to correction (range 0–24), with lower scores reflecting less overconfidence. A composite index (CI) is derived by subtracting SC from SR (CI = SR − SC; range −24 to +36), with higher values indicating better overall cognitive insight. The BCIS was selected because MCT explicitly targets metacognitive functioning, and the BCIS is the most widely used measure of off-line metacognition across MCT efficacy studies. If this self-report instrument raises the question of whether participants with impaired metacognition can accurately evaluate it, the BCIS nonetheless remains the field’s standard tool for assessing this facet of off-line metacognition (Díaz-Cutraro et al., 2026).

### 2.5 Statistical analyses

Prior to the main analyses, group comparability on baseline characteristics was assessed. Baseline BCIS scores were compared between groups using Welch’s t-tests, which are considered more robust than Student’s t-tests regardless of whether variances are equal or unequal (Delacre et al., 2017), and were retained following confirmation of variance homogeneity via Levene’s test. Sex distribution was compared using a chi-squared test.

To examine whether the effect of MCT on cognitive insight was moderated by baseline levels, the ANCOVAs conducted included a continuous Group × Baseline interaction term. Entering baseline as a continuous covariate rather than a stratified variable was preferred for the primary moderation analysis, as retaining the full continuous distribution of baseline scores avoids the information loss and arbitrary threshold dependency inherent to discretization (MacCallum et al., 2002).

As a secondary analysis, participants were stratified into insight severity categories based on their baseline BCIS scores in order to visualize the interaction pattern and compute within-stratum effect sizes. For CI, the cut-offs proposed by Penney et al. (2019) were applied: low (CI ≤ 3), moderate (4 ≤ CI ≤ 9), and high (CI ≥ 10). For SR and SC, no validated clinical thresholds are currently available in the literature. Consistent with the approach used by Penney et al. (2019) to derive their original cut-offs, empirical tertiles were calculated from the distribution of the present sample: tertile 1 (SR ≤ 11), tertile 2 (11 < SR ≤ 16), and tertile 3 (SR > 16) for SR; and tertile 1 (SC ≤ 6), tertile 2 (6 < SC ≤ 9), and tertile 3 (SC > 9) for SC. The representativeness of this approach is supported by the close alignment between the baseline means of the present sample and those reported by Penney et al. (2019) (SR: *M* = 13.88 vs. 13.0; SC: *M* = 7.80 vs. 7.7), confirming the suitability of these thresholds as proxies in the present sample. These categorizations were used for illustrative purposes only and should not be interpreted as clinically validated thresholds. Within each stratum, independent-samples *t*-tests were conducted to estimate Cohen’s *d* effect sizes.

A secondary analysis was conducted to assess the potential influence of the age imbalance between groups, by re-running the ANCOVA models with age entered as an additional covariate, given that age showed group differences at baseline.

To account for potential non-independence of participants within the contributing studies, we conducted a sensitivity analysis using multilevel linear mixed models, with study included as a random intercept, and group, baseline score, and their interaction as fixed effects, applied separately to each of the three BCIS outcomes (SR, SC, CI). Given the relatively small number of studies, the random-effects structure was limited to a random intercept for study, following recommendations favoring random-effect structures that are supported by the data over maximal specifications prone to overparameterization and convergence failure (Matuschek et al., 2017). Reliable estimation of higher-level variance components in multilevel models typically requires a substantially larger number of clusters than available here (Maas and Hox, 2005).

Finally, for completeness, Group × Time comparisons without moderation by baseline severity are reported in Supplementary Table 2 (also see Discussion).

Statistical analyses were performed by pooling all control conditions, as the primary objective was to examine the moderation of MCT efficacy relative to the absence of MCT rather than to compare specific comparator types, consistent with the approach adopted in the broader MCT efficacy literature (Sauvé et al., 2020; Lopez-Morinigo et al., 2020; Goncalves et al., 2025; Meinhart et al., 2025). Analyses were conducted with Jamovi (version 2.7.29) and the figure was generated using RStudio (version 2026.04.0 + 526). Two-tailed significance levels are reported throughout (*p* < 0.05). Given that missing data differed across BCIS subscales, available sample sizes varied by outcome. Analyses for each subscale were conducted on all participants with complete pre- and post-treatment data for that specific subscale, without imputation.

### 2.6 Ethics

Ethical approval for the use of anonymized retrospective data was obtained from the institutional review boards of participating centers. Approval was granted by the Research Ethics Committee of Fundació San Joan de Déu on 27/04/2023 by approval PIC-68-23, the ‘Lokale Psychologische Ethikkomission am Zentrum für Pyschosoziale Medizin’ of UKE on 29/03/2023 by approval LPEK-0603, the ‘Comitè Ético Científico del Servicio de Salud Valparaíso San Antonio’ by approval N∘54/2023 on 27/09/2023, and the ‘Ethics Committee of the UPC’ on 11/12/2023 by approval 2023.13.

Prior to the harmonization process, all data were fully anonymized to protect participant confidentiality. Each study contributing to the retrospective dataset had received ethical clearance from the relevant ethics committees, and written informed consent was obtained from all participants, and their legal guardians where applicable, in accordance with the applicable local regulations. Both the individual studies and the overarching project responsible for the harmonization adhered to the ethical standards set forth in the most recent version of the Declaration of Helsinki.

## 3 Results

### 3.1 Sample characteristics

Table 1 presents descriptive statistics and baseline BCIS scores by treatment group. Of the 614 included participants, 355 received MCT (57.8%) and 259 were allocated to control conditions (42.2%). Diagnostic distribution was as follows: schizophrenia (73.1%), other psychosis (11.7%), schizoaffective disorder (6.1%), brief psychotic disorder (3.4%), delusional disorder (2.5%), schizophreniform disorder (1.7%), and first-episode psychosis (1.5%).

**Table 1:** Sample characteristics and baseline BCIS equivalence by treatment group.

Variable	*MCT* (*n* = 355)	*Control* (*n* = 259)	*t / χ^2^*	*p*
Age M (SD)	36.5 (13.4)	39.7 (12.7)	−3.00	<0.01
Sex Male *n* (%)	229 (64.7)	159 (61.6)	0.53	0.47
SR	14.1 (5.0)	13.6 (5.1)	1.03	0.30
SC	7.8 (3.5)	7.7 (3.4)	0.38	0.70
CI	6.2 (6.4)	5.9 (6.0)	0.72	0.47

SR, Self-Reflectiveness; SC, Self-Certainty; CI, Composite Index; t, Welch’s t-test. Missing data: SR *n* = 6, SC *n* = 9, CI *n* = 14, Sex *n* = 1.

The MCT and control groups differed significantly on age (*p* < 0.01), with MCT participants being on average younger than controls. The MCT and control groups did not differ significantly on sex distribution nor on any of the three baseline BCIS scores (all *p* ≥ 0.30). Levene’s test confirmed homogeneity of variances (SR: *p* = 0.77; SC: *p* = 0.71; CI: *p* = 0.53).

Notably, at baseline, 26.5% of participants scored in the high-insight category on the CI (CI ≥ 10; Penney et al., 2019), indicating that a substantial proportion of the sample already presented with high levels of cognitive insight prior to treatment.

### 3.2 Moderation of MCT efficacy by baseline cognitive insight

When baseline levels were entered as a continuous covariate, ANCOVAs revealed significant Group × Baseline interactions for all three BCIS scores. Stratified ANOVAs yielded significant Group × Baseline interactions for SR and SC, but not for CI (Table 2).

**Table 2:** ANCOVA (Baseline continuous) and ANOVA (Baseline stratified) results by BCIS subscales and composite score.

BCIS score	*F* (Group)	*p*	*η* ^2^ *_p_*	*F* (Baseline)	*p*	*η* ^2^ *_p_*	*F* (Group*Baseline)	*p*	*η* ^2^ *_p_*
	ANCOVA (Baseline continuous)								
SR	7.851	0.**005**	0.016	135.094	**< 0.001**	0.215	5.669	0.**018**	0.011
SC	1.180	0.278	0.002	154.671	**< 0.001**	0.240	7.204	0.**008**	0.014
CI	15.913	**< 0.001**	0.031	130.900	**< 0.001**	0.212	5.579	0.**019**	0.011
	ANOVA (Baseline stratified)								
SR	1.127	0.289	0.002	45.881	**< 0.001**	0.158	4.783	0.**009**	0.019
SC	13.806	**< 0.001**	0.028	55.678	**< 0.001**	0.186	3.654	0.**027**	0.015
CI	42.045	**< 0.001**	0.148	10.418	0.**001**	0.021	2.349	0.097	0.010

SR, Self-Reflectiveness; SC, Self-Certainty; CI, Composite Index. For ANOVAs, SR and SC stratified baseline corresponds to empirical tertiles derived from the present sample (T1: SR ≤ 11, T2: 11 < SR ≤ 16, T3: SR > 16; T1: SC ≤ 6, T2: 6 < SC ≤ 9, T3: SC > 9). For CI, stratified baseline corresponds to Penney et al. (2019) cut-offs (Low ≤ 3, Moderate 4–9, High ≥ 10). Bold values indicate statistical significance (*p* < 0.05).

To visualize this interaction pattern and estimate within-stratum effect sizes, participants were stratified into baseline insight categories. The results reveal a consistent gradient pattern across baseline insight categories. For SC, where more negative values reflect greater reduction in overconfidence, effect sizes became increasingly negative from tertile 1 (*d* = −0.025) to tertile 3 (*d* = −0.520), with significant effects in tertiles 2 and 3 only. For SR and CI, where higher values reflect greater insight improvement, a similar inverse pattern was observed, with the largest significant effect in tertile 1 (*d* = 0.505; *d* = 0.583, respectively) and negligible, non-significant effects in tertiles 2 (*d* = −0.136; *d* = 0.160, respectively) and 3 (*d* = −0.061; *d* = 0.158, respectively), as illustrated in Figure 1.

**Figure 1:**
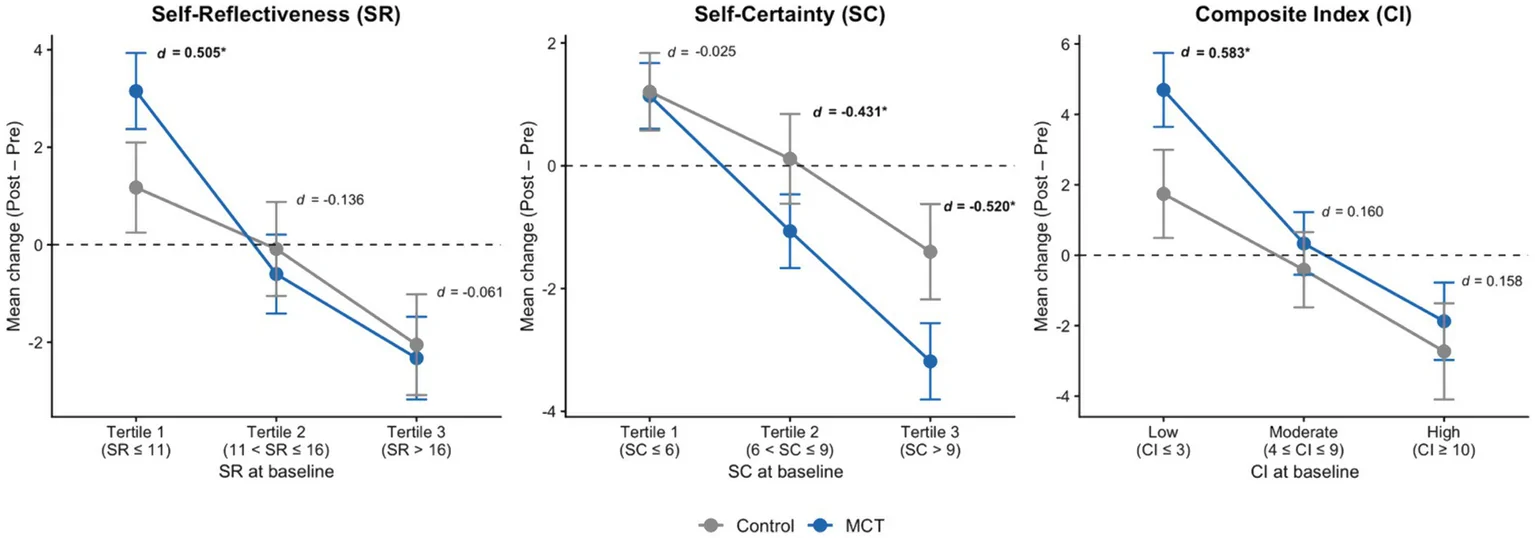
Mean pre-to-post changes in SR, SC, and CI of the BCIS by treatment group and baseline insight category. Error bars represent 95% confidence intervals. Stratification for SR and SC correspond to empirical tertiles derived from the present sample (T1: SR ≤ 11, T2: 11 < SR ≤ 16, T3: SR > 16; T1: SC ≤ 6, T2: 6 < SC ≤ 9, T3: SC > 9). Stratification for CI correspond to Penney et al. (2019) cut-offs (Low ≤ 3, Moderate 4–9, High ≥ 10). Cohen’s d effect sizes are reported for each category. *p < 0.05.

When age was included as an additional covariate in the secondary ANCOVA models, the Group × Baseline interaction remained significant across all three BCIS scores (SR: *F* = 5.510, *p* = 0.019, *η*^2^*_p_* = 0.011; SC: *F* = 7.210, *p* = 0.007, *η*^2^*_p_* = 0.015; CI: *F* = 5.370, *p* = 0.021, *η*^2^*_p_* = 0.011), and age did not emerge as a significant predictor in any model (all *p*s ≥ 0.287).

### 3.3 Sensitivity analysis

A sensitivity analysis using multilevel linear mixed models with study included as a random intercept converged without warnings for all three outcomes and yielded a pattern of results consistent with the primary ANCOVA models. The Group × Baseline interaction remained significant for SR (*b* = 0.165, SE = 0.069, 95% CI [0.031, 0.300], *p* = 0.016), SC (*b* = 0.193, SE = 0.074, 95% CI [0.049, 0.338], *p* = 0.009), and CI (*b* = 0.170, SE = 0.069, 95% CI [0.034, 0.306], *p* = 0.015), indicating that the moderating effect of baseline cognitive insight was robust to accounting for between-study variability.

Unmoderated Group × Time comparisons are also reported in Supplementary Table 2 for information.

## 4 Discussion

This study examined whether the efficacy of MCT on cognitive insight varies according to baseline BCIS scores. ANCOVAs revealed significant Group × Baseline interactions across all BCIS dimensions, and stratified ANOVAs yielded convergent results for SR and SC, collectively indicating that baseline levels are associated with variation in treatment effects. Importantly, effect sizes followed a clear gradient: participants with greater baseline impairment derived greater benefit from MCT. Taken together, these findings suggest that MCT may produce clinically meaningful improvements in cognitive insight, particularly among participants presenting with the most pronounced baseline impairment.

These results should be interpreted in light of a methodological observation that may have contributed to the inconsistency of effects on cognitive insight reported in previous studies (Lopez-Morinigo et al., 2020; Sauvé et al., 2020; Goncalves et al., 2025). At baseline, 26.5% of participants already scored in the high-insight category on the CI, indicating that a substantial proportion of the sample presented with no clinically meaningful impairment on this dimension prior to treatment. This may constitute a ceiling effect that likely attenuated treatment effects at the group level (Hegedus and Moody, 2010; Andrade, 2021). This ceiling effect may reflect the fact that cognitive insight has rarely, if ever, been a primary outcome in MCT efficacy trials, which would explain why no minimum cognitive insight thresholds have generally been implemented as inclusion criteria in these studies.

The validity of this ceiling-effect interpretation is supported by several methodological strengths of the present study. The use of individual participant data from 13 international studies, representing a sample of 614 participants, ensures robust statistical power and cross-cultural generalizability. Confidence in the observed moderation pattern is further strengthened by the convergence of results across three analytical approaches with distinct assumptions and statistical properties: the continuous ANCOVA, the stratified ANOVA, and a multilevel mixed model accounting for between-study clustering, which yielded a virtually identical pattern of results across all three BCIS scores. This aligns with the framework advocated by Munafò and Davey Smith (2018), which reduces the risk that the observed patterns reflect methodological artifacts rather than genuine moderation effects. Minor discordances between the two approaches also warrant brief comment. The Group × Baseline interaction for CI was significant in the continuous ANCOVA but not in the stratified ANOVA, which may be consistent with the expected loss of power inherent to discretizing a continuous moderator (MacCallum et al., 2002), further supporting the *a priori* decision to treat the continuous ANCOVA as the primary approach. Similarly, the Group main effect for SR and SC diverged in significance between the two models; this may partly reflect differences in how each model estimates the group effect across the distribution of baseline scores, and may illustrate how averaging across participants with heterogeneous baseline profiles could obscure genuine treatment effects, potentially accounting for the inconsistent findings reported in the existing literature (Lopez-Morinigo et al., 2020; Goncalves et al., 2025; Houdebine et al., 2025). Furthermore, although MCT and control groups differed significantly on age at baseline, secondary analyses including age as additional covariate yielded virtually identical results across all three BCIS scores. This is consistent with recent large-scale meta-analyses indicating that age does not moderate MCT efficacy (Penney et al., 2022; Gelner et al., 2025).

Several limitations warrant consideration. First, the present analysis did not examine other potential moderators of the baseline severity-efficacy relationship, such as positive symptom severity, illness duration, or self-esteem (König et al., 2025a). Second, the present findings should not be generalized to MCT’s effects on metacognition more broadly, as metacognitive abilities extend well beyond cognitive insight as operationalized by the BCIS, which captures specifically the metacognitive awareness component of off-line metacognition (Díaz-Cutraro et al., 2026). Other metacognitive self-report measures were available in only a handful of isolated datasets, precluding harmonization across studies (Goncalves et al., 2025), and a complementary hetero-report or performance-based assessment could not be implemented here, although recommended for research settings (Díaz-Cutraro et al., 2026). Moreover, as a self-report measure, the BCIS raises the epistemological question of whether individuals with impaired metacognitive abilities are well-positioned to accurately evaluate the very capacities that are by definition compromised (Bröcker et al., 2017). This limitation should nevertheless be distinguished from the broader question of whether BCIS scores contain meaningful information. Previous findings suggest that its subscales show distinct associations with clinically and cognitively relevant outcomes: lower baseline self-certainty predicts greater objective cognitive gains after remediation (Benoit et al., 2016), whereas self-reflectiveness, unlike self-certainty, prospectively predicts symptom severity independently of objective cognitive measures (O’Connor et al., 2017). More broadly, self-assessment of cognitive functioning in schizophrenia appears heterogeneous rather than uniformly impaired (Raffard et al., 2020). Thus, while these considerations do not eliminate the epistemological concern inherent in the self-report assessment of impaired metacognition, they support a cautious interpretation of BCIS scores rather than their wholesale dismissal. Third, the scarcity of follow-up data across contributing studies precluded a systematic examination of the durability of the observed effects, as evidence suggests that the effects of MCT on clinical outcomes may evolve over time, with both consolidation and attenuation patterns reported at follow-up (Lopez-Morinigo et al., 2020; Penney et al., 2022; Goncalves et al., 2025). Fourth, the 13 contributing studies represent only a subset of available MCT trials, potentially biased by the willingness of research teams to share data. They may not be entirely representative of all MCT research. This concern is compounded by the considerable heterogeneity across contributing studies in terms of MCT format, number of sessions, control condition type, and clinical setting, which may have introduced additional variability in treatment response. However, this heterogeneity in MCT format was largely confounded with study-level differences, precluding its formal testing as a moderator of treatment efficacy in the present analysis. Future research directly comparing different MCT formats within the same study would be needed to disentangle format effects from study-level differences. Fifth, because the present analysis was designed *a priori* to test whether baseline cognitive insight moderates MCT efficacy, rather than to establish overall treatment efficacy independent of baseline severity, a question already addressed by existing meta-analytic evidence (Lopez-Morinigo et al., 2020; Sauvé et al., 2020), unmoderated group comparisons are not reported in the main text. For transparency, these are provided in Supplementary Table 2 and reached significance for SC and CI but not for SR. Finally, although statistically significant Group × Baseline interactions were observed, the absolute magnitude of the between-group differences in BCIS scores was modest, and the clinical significance of changes of this magnitude in daily functioning or symptom outcomes remains to be established. Future studies should examine the relationship between MCT-induced changes in cognitive insight and concurrent changes in psychotic symptoms or daily functioning, which would allow for a more direct assessment of the clinical relevance of improvements in this domain.

Given the prognostic and functional significance of cognitive insight outlined in the Introduction, the present findings carry implications for both clinical practice and future research. From a clinical standpoint, the gradient of efficacy observed across baseline cognitive insight levels suggests that participants presenting with the most pronounced impairments, characterized by low self-reflectiveness and high self-certainty, may particularly benefit from MCT when improving metacognitive functioning is a shared therapeutic goal. This finding is all the more relevant given that schizophrenia remains among the most highly stigmatized mental disorders, including among mental health professionals (Valery and Prouteau, 2020), and that clinicians themselves report defaulting to a deficit-focused view of cognitive functioning as standard practice (Steele et al., 2021). Within such a framework, there is a risk that MCT may be preferentially offered to individuals perceived as sufficiently cognitively able to engage with the intervention, whereas the present findings suggest that those with more pronounced metacognitive difficulties may have greater room for improvement. At the same time, using baseline cognitive insight to identify such difficulties should not reinforce an exclusively deficit-oriented characterization of participants. Rather, its clinical use should be embedded within a recovery-oriented approach that remains attentive to the potential consequences of stigma and to the preservation of self-esteem. Indeed, greater metacognitive capacity has been associated with stronger resistance to internalized stigma (Nabors et al., 2014), while the negative association between internalized stigma and self-esteem has been shown to be mediated by off-line metacognitive complaints (Violeau et al., 2020). Accordingly, identifying metacognitive difficulties may be clinically useful not only for informing the indication of MCT, but also for guiding how these difficulties are discussed with participants. From a research perspective, these results advocate for the systematic inclusion of baseline cognitive insight measures in future MCT trials, and for the consideration of minimum impairment thresholds when cognitive insight constitutes a primary outcome. More broadly, the pattern observed here is consistent with findings from a parallel individual participant data analysis from our group examining MCT’s efficacy on positive symptoms taking baseline clinical severity into account (Goncalves et al., under review). Future studies should examine whether combining baseline positive symptom severity and cognitive insight profiles can further refine the identification of participants most likely to demonstrate measurable gains on these specific outcomes in efficacy trials, rather than informing treatment eligibility, in line with precision medicine approaches to psychological treatment of psychosis (König et al., 2025b; Lamarca et al., 2026).

## 5 Conclusion

Baseline levels of cognitive insight moderate the efficacy of MCT, with participants presenting the greatest impairment at baseline deriving the largest benefit. A non-negligible proportion of participants already scored in the high-insight category at baseline, suggesting that ceiling effects may have partially reduced the positive treatment effects of MCT on cognitive insight in previous trials. Accounting for baseline heterogeneity may clarify inconsistent findings across studies and improve the interpretation of cognitive insight outcomes.

## The group members of the PERMEPSY group

The authors would like to acknowledge all PERMEPSY members: Susana Ochoa, Maria Lamarca, Belen Ramos Josemaria, Judith Usall, Regina Vila Badia, Raquel Lopez Carrilero, Trini Pelaez Martinez, Irene Birulés, and Claudia Requejo; Partner 1 members Steffen Moritz, Rabea Fischer, Merle Schlechte, Annika Schmueser, Antonia Meinhart, and Jakob Scheunemann; Partner 2 members Fabrice Berna and Adrien Goncalves; Partner 3 members Łukasz Gawęda, Marytna Krezolek, Hania Gelner, Adrianna Aleksandrowicz, and Justyna Piwinska; Partner 4 members Caroline König, Pedro Copado, Àngela Nebot, Alfredo Vellido, Cecilio Angulo, and Marc Larroda; and Partner 5 members Vanessa Acuña and Alvaro Cavieres.

## Data Availability

The PERMEPSY harmonized dataset underlying this study is not publicly available due to ethical and data governance constraints across the contributing international studies. Researchers wishing to access the dataset may submit a formal request to the PERMEPSY consortium (www.permepsy.org), where requests are reviewed by the steering committee in accordance with applicable ethical guidelines. The data harmonization pipeline and variable structure are fully documented in König et al. (2025b) and the associated Python scripts are publicly available at https://gitlab-rdlab.cs.upc.edu/soco-permepsy/harmonization. The complete Jamovi analysis template (without patient data) has been deposited on the Open Science Framework: https://osf.io/a8yf7/overview?view_only=a1a1ac93340543058ca396ee75aa3b4e. Requests to access these datasets should be directed to www.permepsy.org. Any further inquiries can be directed to the corresponding author.
