# Supplementary material for: Baseline cognitive insight moderates the efficacy of metacognitive training for psychosis: an individual participant data analysis
Source: Front Psychol. 2026 Oct 27;17:1934181. doi: 10.3389/fpsyg.2026.1934181 (PMC13748317; doi:10.3389/fpsyg.2026.1934181)
Supplement: Supplementary file 1 [file Supplementary_file_1.pdf]

Supplementary Table 1. Characteristics of the 22 studies included in the PERMEPSY database.

Main author	DOI	Year of publication	Country	Center	Setting	Diagnosis	Sex (♂ - ♀)	MCT Format (participants)	Controls (participants)	MCT sessions (Max)	BCIS available
Acuña	Unpublished	-	Chile	-	Outpatients	SCZ	29 - 17	MCT (46)	-	10	No
Balzan*	10.1093/schbul/sby152	2019	Australia	Mono	Outpatients	SSD	32 - 22	MCT+ (27)	CR (27)	4	Yes
Favrod	10.1016/j.jtcc.2015.06.002	2015	Switzerland	Multi (2)	Outpatients	SSD	34 - 18	MCT (26)	TAU (26)	8	No
Fujii*	10.11596/asiajot.17.45	2021	Japan	Mono	-	SSD	10 - 7	MCT (9)	OT (8)	16	Yes
Gawęda	Unpublished	-	Poland	-	In and outpatients	NS	23 - 25	MCT (25)	Discussion (23)	NA	No
Ishikawa*	10.1016/j.schres.2019.08.006	2020	Japan	Multi (6)	In and outpatients	SSD	25 - 25	MCT (24)	TAU (26)	10	Yes
Kuokkanen*	10.1002/cbm.1905	2014	Finland	Mono	Inpatients *(Forensic)*	SCZ	10 - 0	MCT (10)	-	8	Yes
Leanza	10.1016/j.jbtep.2020.101547	2020	Germany	Mono	In and outpatients	SSD	51 - 41	MCT+ (46)	CR (46)	12	No
Lopez-Morinigo*	10.1038/s41537-022-00316-x	2023	Spain	Mono	Outpatients	SSD	41 - 36	MCT (39)	PE (38)	10	Yes
Moritz 1*	10.1017/S0033291710002618	2011	Germany	Mono	Inpatients	SCZ	31 - 17	MCT+ (24)	CR (24)	8	Yes
Moritz 2	10.1016/j.schres.2013.10.007	2013	Germany	Multi (2)	In and outpatients	SSD	97 - 56	MCT (79)	CR (74)	8-16	No
Moritz 3*	10.1016/j.brat.2010.11.010	2011	Germany	Multi (2)	Outpatients	SSD	28 - 8	MCT (18)	TAU (18)	8	Yes
Ochoa 1*	10.1017/S0033291716003421	2017	Spain	Multi (9)	-	SSD	85 - 37	MCT (65)	PE (57)	8	Yes
Ochoa 2*	NA	2020	Spain	Multi (10)	-	SSD	44 - 25 (1 NA)	MCT+ (36)	TAU (34)	10	Yes
Ochoa 3	Unpublished	-	Spain	-	-	SSD	28 - 22 (4 NA)	MCT (54)	-	8-10	Yes
De Pinho*	10.1111/jan.14627	2020	Portugal	Multi (4)	In and outpatients	SCZ	30 - 22	MCT (26)	TAU (26)	8	Yes
Simon-Exposito*	10.3390/ijerph16224541	2019	Spain	Multi (2)	Inpatients	SSD	16 - 6	MCT (11)	TAU (11)	16	Yes
So	10.3389/fpsyg.2015.00730	2015	China	Mono	Outpatients	SSD	24 - 20	MCT+ (23)	TAU (21)	4	No
Swanson	10.1002/cpp.2692	2021	Scotland	Mono	In and outpatients	SSD	13 - 2	MCT-N (15)	-	8	No

Tanoue*	Unpublished	-	Japan	-	Outpatients	SCZ	14 - 8	MCT (22)	-	NA	Yes
Ussorio*	10.1111/papt.12059	2016	Italy	Mono	Outpatients *(Early intervention)*	SSD	30 - 13	MCT Young Version (43)	-	16	Yes
Yildiz	10.29399/npa.23095	2019	Turkey	Mono	Outpatients	SSD	13 - 7	MCT (10)	PST (10)	40	No

Note. *MCT = MetaCognitive Training (group); MCT+ = MetaCognitive Training (individual); MCT-N = MetaCognitive Training (group) - variant targeting negative symptoms; CR = Cognitive remediation; OT = Occupational therapy; PE = Psychoeducation; PST = Psychosocial Skills Training; TAU = Treatment as usual; SSD = Schizophrenia spectrum disorder; SCZ = Schizophrenia; NA = Not available; *Study retained in the present cognitive insight analysis (see Methods, Section 2.1).* This table is *Adapted from Goncalves et al. (2026). Schizophr. Res. Cogn. 45:100440. https://doi.org/10.1016/j.scog.2026.100440 (CC BY 4.0).*

Supplementary Table 2. Repeated-measures ANOVA (Group × Time) results for BCIS subscales and composite score, without accounting for baseline severity.

	Repeated-measures ANOVA								
	F (Group)	***p***	η^2_p	***F*** (Time)	***p***	η^2_p	***F*** (Group*Time)	***p***	η^2_p
**SR**	2.201	.139	.004	0.003	.958	<.001	1.061	.303	.002
**SC**	0.591	.442	.001	4.862	**.028**	.010	10.226	**.001**	.020
**CI**	2.487	.115	.005	1.918	.167	.004	7.622	**.006**	.015

Note. SR = Self-Reflectiveness; SC = Self-Certainty; CI = Composite Index. Bold values indicate statistical significance (p < .05).
